# Radiology artificial intelligence: a systematic review and evaluation of methods (RAISE)

**DOI:** 10.1007/s00330-022-08784-6

**Published:** 2022-04-14

**Authors:** Brendan S. Kelly, Conor Judge, Stephanie M. Bollard, Simon M. Clifford, Gerard M. Healy, Awsam Aziz, Prateek Mathur, Shah Islam, Kristen W. Yeom, Aonghus Lawlor, Ronan P. Killeen

**Affiliations:** 1 St Vincent’s University Hospital, Dublin, Ireland; 2 Insight Centre for Data Analytics, UCD, Dublin, Ireland; 3 Wellcome Trust – HRB, Irish Clinical Academic Training, Dublin, Ireland; 4 School of Medicine, University College Dublin, Dublin, Ireland; 5 Lucille Packard Children’s Hospital at Stanford, Stanford, CA USA; 6 HRB-Clinical Research Facility, NUI Galway, Galway, Ireland; 7 Division of Brain Sciences, Imperial College London, GN1 Commonwealth Building, Hammersmith Hospital, Du Cane Road, London, W12 0HS UK

**Keywords:** Radiology, Artificial Intelligence, Methodology, Systematic reviews

## Abstract

**Objective:**

There has been a large amount of research in the field of artificial intelligence (AI) as applied to clinical radiology. However, these studies vary in design and quality and systematic reviews of the entire field are lacking.This systematic review aimed to identify all papers that used deep learning in radiology to survey the literature and to evaluate their methods. We aimed to identify the key questions being addressed in the literature and to identify the most effective methods employed.

**Methods:**

We followed the PRISMA guidelines and performed a systematic review of studies of AI in radiology published from 2015 to 2019. Our published protocol was prospectively registered.

**Results:**

Our search yielded 11,083 results. Seven hundred sixty-seven full texts were reviewed, and 535 articles were included. Ninety-eight percent were retrospective cohort studies. The median number of patients included was 460. Most studies involved MRI (37%). Neuroradiology was the most common subspecialty. Eighty-eight percent used supervised learning. The majority of studies undertook a segmentation task (39%). Performance comparison was with a state-of-the-art model in 37%. The most used established architecture was UNet (14%). The median performance for the most utilised evaluation metrics was Dice of 0.89 (range .49–.99), AUC of 0.903 (range 1.00–0.61) and Accuracy of 89.4 (range 70.2–100). Of the 77 studies that externally validated their results and allowed for direct comparison, performance on average decreased by 6% at external validation (range increase of 4% to decrease 44%).

**Conclusion:**

This systematic review has surveyed the major advances in AI as applied to clinical radiology.

**Key Points:**

*• While there are many papers reporting expert-level results by using deep learning in radiology, most apply only a narrow range of techniques to a narrow selection of use cases.*

*• The literature is dominated by retrospective cohort studies with limited external validation with high potential for bias.*

*• The recent advent of AI extensions to systematic reporting guidelines and prospective trial registration along with a focus on external validation and explanations show potential for translation of the hype surrounding AI from code to clinic.*

**Supplementary Information:**

The online version contains supplementary material available at 10.1007/s00330-022-08784-6.

## Introduction

Artificial intelligence (AI) applications in radiology are gaining more and more attention. Recently, the performance of deep learning (DL) on computer vision (CV) tasks has revolutionised the field. Specifically, since the increase in performance in the ImageNet challenge by Hinton and colleagues with AlexNet [1], a convolutional neural network (CNN), there has been interest in potential applications in the medical field. Radiology as a digital image-based speciality was touted as an early potential testing ground for medical applications of CV [2]. This, coupled with an increasing demand for clinical imaging and an international shortage of radiologists [3], drove interest. Radiology conferences and journals have seen a large increase in AI-based submissions [4], and new journals [5] have even been established to keep pace with the increase in the literature.

However, the initial hype may be beginning to come to an end. Dos Santos and colleagues have warned about the possibility of a reproducibility crisis and translation gap in radiomics [6] and many of these issues also exist in DL. While radiology does lead the way in medical AI device approval [7], the gap between the promise in the literature and the clinical application of these models has been termed the “AI Chasm” by Topol and Keane [8]. The gap is due in part to the models being tested in “ideal” in silico conditions and therefore not having robust performance in the clinic [9]. Furthermore, the generalizability of models from the home institution to others has proved an issue [10]. As models become more complex to account for the above difficulties, their outputs become less interpretable and a lack of explicability has led to worries of potential bias. Furthermore, as the nature of the field is multidisciplinary, the difficulty in forming research teams with combined clinical, radiological, engineering and computer science expertise may prove a challenge [11]. This is compounded by the need for new expertise in the peer review process [4]. As such, while the volume of literature has increased, the quality is varied.

There have been calls for high-level evidence especially prospective studies and outcomes data [6]. To this end, this systematic review aims to survey the literature to identify the clinical questions being asked and the methods used to answer them, with a focus on the scientific methodology underpinning these studies. We will report the state of radiology AI both to assess the quality of the existing data and identify potential opportunities for further research.

## Methods

We followed the Preferred Reporting Items for Systematic Review and Meta-Analysis guidelines (PRISMA) and the Cochrane Collaboration Handbook and performed a systematic review of all radiology AI studies published from 2015 to 2019 following a published protocol of a prospectively registered review (PROSPERO: CRD42020154790).

Full details are available in the published protocol [12].

### Inclusion criteria

This comprehensive review includes all clinical radiological papers that aim to complete a task using DL. Human hospital-based studies that use these techniques to aid in the care of patients’ radiological diagnosis or intervention are included. Studies based on radiographic, computed tomography (CT), magnetic resonance (MR), ultrasound (US) or nuclear medicine/molecular or hybrid imaging techniques are included.

### Exclusion criteria

Studies that use classical machine learning techniques are excluded. Functional MRI (fMRI) papers are not included as the techniques used in the computer analysis of fMRI data are quite separate from the computer vision–based tasks that are the subject of this review. Papers solely for use in radiation therapy are also excluded. Non-human or phantom studies are excluded.

### Electronic search

We performed electronic searches on MEDLINE (Pubmed) and EMBASE from 1 January 2015 until 31 December 2019. Zotero was our reference manager and the Revtools package in R was used to eliminate duplicate records. The search was conducted in English. The search terms used are reported in the Supplementary Appendix. The Artificial Intelligence and Radiology terms were combined with the ‘AND’ operator.

### Selection and analysis of trials

**Fig. 1:**
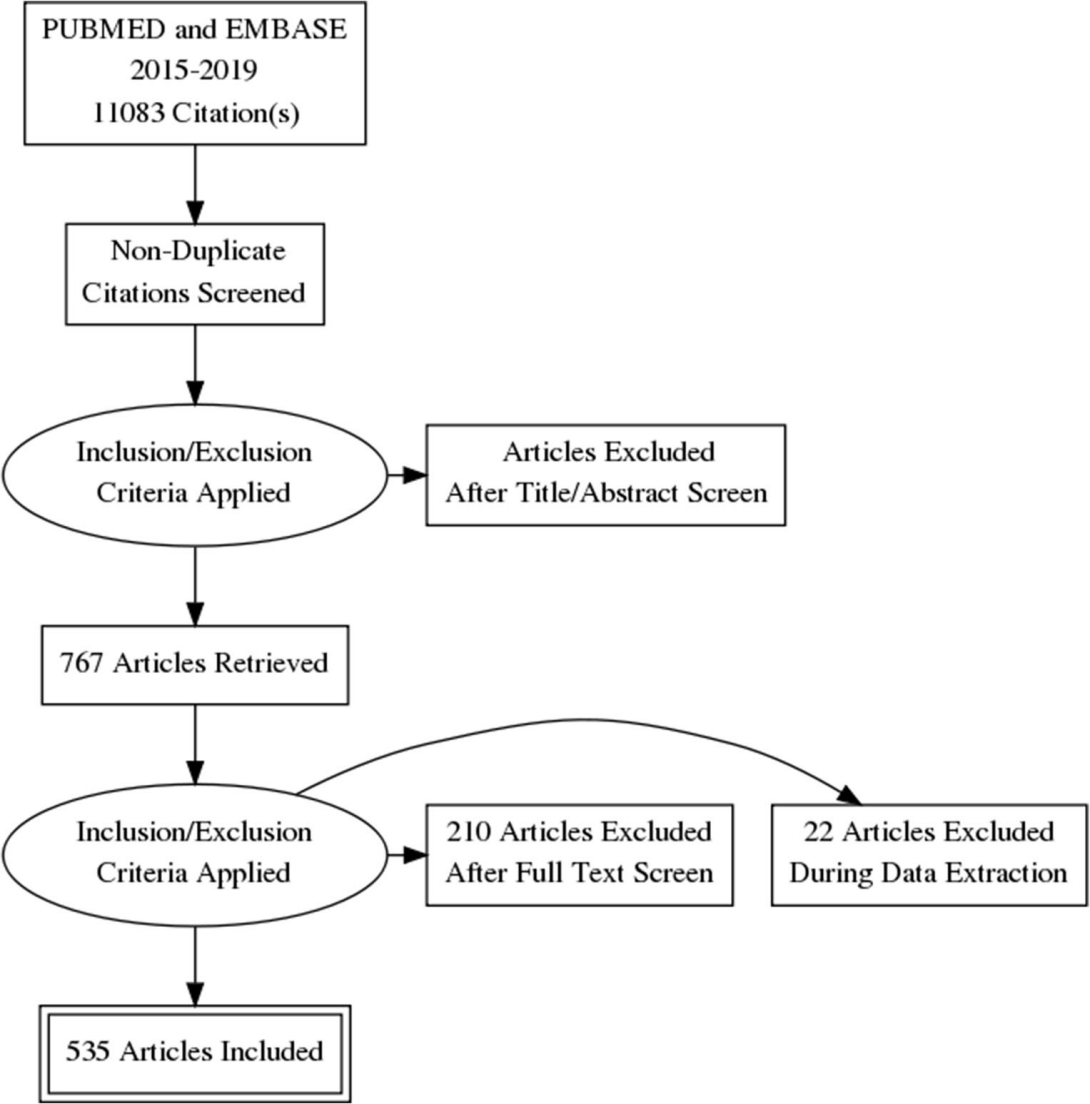
The PRISMA flow diagram of papers included in our review

The titles and abstracts of studies were reviewed to identify clinical radiological artificial intelligence studies for inclusion or exclusion. Studies with insufficient information to determine the use of AI computer vision methods were also included for full-text review. A full-text review was then performed, to confirm eligibility for inclusion in the final systematic review. This process is summarised in a PRISMA flowchart (Fig. 1).

### Data analysis

Data were analysed primarily using pivot tables and in-built exploratory analysis tools in Microsoft Excel. A narrative synthesis was undertaken due to the heterogeneity of methodologies employed.

## Results

### Included papers

Our search yielded 11,083 results. Titles and abstracts were screened by two reviewers and 767 full texts were reviewed. Five hundred thirty-five articles were included for analysis. Details can be found in Fig. 1.

**Fig. 2:**
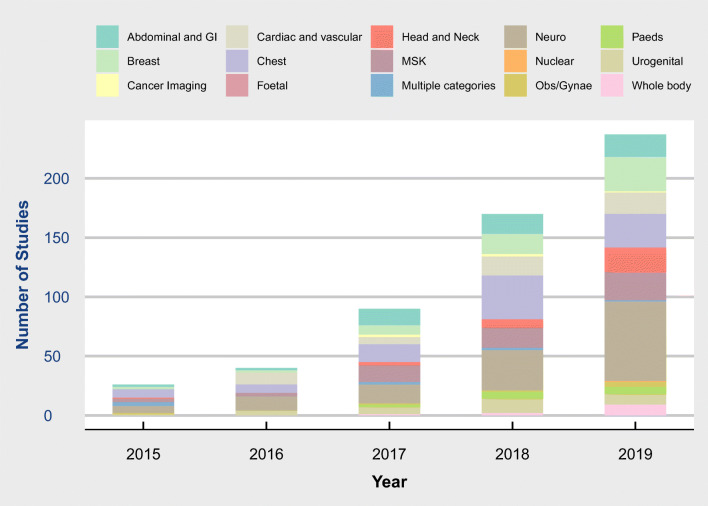
Radiology artificial intelligence articles by clinical area and year

**Fig. 3:**
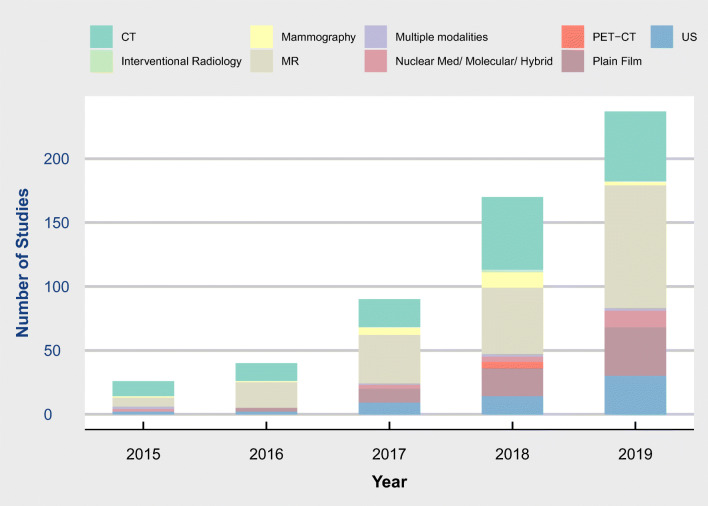
Radiology Artificial Intelligence Articles by modality and year. Modality investigated by year. Colour denotes modality

There was an increase in the number of included studies year on year with 14, 31, 84, 170 and 237 respectively from 2015 to 2019 (Figs. 2 and 3).

### Clinical use cases

Cancer imaging was the most common use case, seen in 156 (29%) studies with a further 45 (8%) studies specifically examining pulmonary nodules. The next most common was segmentation of normal anatomy or other investigation of patients with no specific disease (Table 1).

**Table 1:** Legend search terms combined with the AND operator

Artificial intelligence	Radiology
(Artificial intelligence[Title/Abstract])OR (Machine learning[Title/Abstract])OR (Support vector machine[Title/Abstract])OR (SVM[Title/Abstract])OR (CNN[Title/Abstract])OR (RNN[Title/Abstract])OR (LSTM[Title/Abstract])OR (ResNet[Title/Abstract])OR (DenseNet[Title/Abstract])OR (Unet[Title/Abstract])OR (U-net[Title/Abstract])OR (DNN[Title/Abstract])OR (Neural network*[Title/Abstract])OR (Convolutional network*[Title/Abstract])OR (Deep learn*[Title/Abstract])OR (Semantic segmentation[Title/Abstract])OR (Ensemble[Title/Abstract])OR (Classification tree[Title/Abstract])OR (regression tree[Title/Abstract])OR (probability tree[Title/Abstract])OR (nearest neighbo*[Title/Abstract])OR (fuzzy logi*[Title/Abstract])OR (random forest[Title/Abstract])OR (kernel[Title/Abstract])OR (k-means[Title/Abstract])OR (naive bayes[Title/Abstract])	(X-ray*[Title/Abstract])OR (X-ray*[Title/Abstract])OR (Radiography[Title/Abstract])OR (Radiograph*[Title/Abstract])OR (Computed tomography[Title/Abstract])OR (CT[Title/Abstract])OR (CAT[Title/Abstract])OR (CTA[Title/Abstract])OR (Computerized axial tomography[Title/Abstract])OR (Magnetic resonance imag*[Title/Abstract])OR (MRI[Title/Abstract])OR (MR[Title/Abstract])OR (Magnetic resonance angio*[Title/Abstract])OR (MRA[Title/Abstract])OR (Scintigraphy[Title/Abstract])OR (DMSA[Title/Abstract])OR (Ultrasound*[Title/Abstract])OR (Sonograph*[Title/Abstract])OR (PET[Title/Abstract])OR (Positron Emission Tomography[Title/Abstract])OR (SPECT[Title/Abstract])OR (Single-photon emission[Title/Abstract])OR (Single photon emission[Title/Abstract])OR (mammogra*[Title/Abstract])

Trauma, Alzheimer’s and neurodegenerative disease, stroke, coronary artery disease, pneumonia and haemorrhage combined made up 70 (13%) studies with the remainder coming from a variety of use cases (Table 1).

The most common subspecialty was neuroradiology (127, 24%) followed by chest (92, 17%) (Fig. 2). Cross-sectional imaging was dominant with MR (200, 37%) and CT (155, 29%) used as the modality of investigation (Fig. 3).

### Artificial intelligence task

The most common primary task undertaken was segmentation (211, 39%), followed by classification (171, 31%) and identification (74) (Table 1). A further 36 were a combination of those use cases. Twenty-seven studies were primarily aimed at prediction with a further 3 studies having a prediction element to the research question. The remaining studies examined change detection, time series and regression problems.

### Clinical Research Design

Five hundred twenty-five (98%) studies were retrospective with only 13 prospective studies. Five hundred twenty (97%) were designed as cohort studies with 9 case-control studies, 6 designed to measure AI augmentation of performance and 1 with prospective real-world evaluation. A power analysis was referenced in 29 (5%) cases. Inclusion and exclusion criteria were provided in 468 (87%) cases.

Ground truth was determined from the radiologic report in 355 (66%) cases and by a panel of experts or other speciality reports in 100 (19%) cases. A pathologic report determined ground truth in 46 cases (9%). An alternative modality reported by a radiologist was used in 4 cases. Truth determination was unclear in 3 cases.

The performance comparison was a state-of-the-art model in 197 (37%) cases, radiologist(s) in 169 (31%), other medical experts in 28 and a combination of radiologists and other medical experts in 16. There was no comparison in 92 (17%) cases. Comparisons with non-experts including residents, radiographers, sonographers and medically naïve humans made up the remainder.

### Artificial intelligence methodology

One hundred fifty-six (29%) studies used a custom deep learning architecture. UNet was the most popular established architecture used in 76 (14%), followed by ResNet. Ensemble methods were described in 19 (4%) cases. Where the model was previously described (313), it was modified in 275 (88%) cases and used “off the shelf” in the remainder.

Supervised learning was employed in 473 (88%) studies, unsupervised learning in 13, a combination of both in 8 and semisupervised learning in 6. Methods were unclear in 38 (7%) cases. Transfer learning was not used in 284 (53%), was used in 247 (46%) and was unclear in the remainder.

The primary evaluation metric was Dice in 187 (35%) cases (segmentation), area under curve (AUC) in 154 (29%), accuracy in 82 (15%), sensitivity and specificity in 40 (diagnostic accuracy), correlation in 18, mean absolute error in 10 (regression) and precision/recall in 8 (diagnostic accuracy).

Data augmentation was not undertaken in 124 (23%) studies. Most studies used simple operations such as symmetry operations zooms and flips (*n* = 325) with more complex augmentation, e.g. with Generative Adversarial Networks used less frequently (*n* = 31).

In 400 (75%) papers, it was clear that the authors had employed hyperparameter optimisation. It was not undertaken in 65 (12%) and unclear in the remainder.

### Data

The median number of patients included was 460 (range 3–313,318). The median number of images (where reported) was 1993 (range 43–1,351,090). There were on average just over 4 images per patient. The median number of cases (points in time) was 488, giving an average of just over 1 case per patient. Data were sourced from one hospital in 234 (44%) cases, a public dataset in 170 cases (32%), more than one hospital in 67(12%) and public and local data combined in 49 cases (22%). It was unclear in the remaining cases. Results were validated in 352 cases. External validation was used in 169 (31%) with 60 of these using public data for external validation. Of the 109 studies validated at external institutions, 84 were in 2018 and 2019 (78%).

### Performance

The median performance for the most common evaluation metrics was Dice of 0.89 (range .49–.99), AUC of 0.903 (range 1.00–0.61) and accuracy of 89.4 (range 70.2–100).

Comparison of the most commonly used previously described model (U-Net) with custom-made architectures by the Dice metric (*n* = 106.57 U-Net and 49 custom) showed very similar performance (0.877 and 0.895).

Of the 109 studies that externally validated their results, a direct comparison between internal and external performance was possible in 77 cases. The performance on average decreased by 6% at external validation (range 4% improvement to 44% reduction). Sixty of 77 (78%) studies reported a drop of performance of 10% or less.

### Open access data and code

The source code or model was made freely available in 76 cases (14%). The data used is available freely in 207 (38%) studies and a subset of the data is available in 25 (5%).

### Explainability

The most common method of explainability was the provision of cases/examples seen in 263 (49%) studies. No explainability was offered in 152 (28%) cases. Visualisations and saliency maps were offered as explanations in 97 (18%) and there were analytic/didactic discussions in 26 (5%). Counterfactual examples were not employed in any case.

## Discussion

### Overview

This systematic review aimed to survey the literature in radiology artificial intelligence. In doing so, we hoped to identify the clinical questions being asked and the methods used to answer them. We chose to focus on the scientific methodology underpinning included studies. We identified over 500 relevant studies, the number of which is exponentially increasing year on year. We identified that most studies are focused on a narrow range of research questions dominated by segmentation of normal anatomy and classification of tumours and nodules, with a focus on Neuroradiology and MR imaging. The majority of studies applied supervised learning to retrospective data.

The potential for the use of AI to meet the supply-demand issue caused by the increasing utilisation of medical imaging and the international shortage of expert radiologists is well established. We have demonstrated through our review, however, that the majority of the literature uses a narrow range of techniques and focuses on only a few clinical questions. As such, if the “AI chasm” is to be bridged, shortcomings in the implementation of AI clinically will need to be overcome. This also points to opportunities for future research in more diverse methodologies and application to novel use cases.

### Narrow focus

The most common use cases identified were segmentation and classification of tumours and nodules. The focus on tumour imaging was stark with almost 10% of papers specifically examining pulmonary nodules. While papers may have focused on different types of tumours, the basic methodology of identification, segmentation and classification of a lesion in an image dominate the literature. Very few studies considered change between studies or attempted to predict the clinical course of the patient. There were no studies with outcomes data.

As literature is also dominated by segmentation, the “U-Net” [13] architecture is the most commonly employed model. Interestingly, only a modest improvement was seen between using U-Net compared to a custom architecture. The emphasis on segmentation, identification and classification has meant that alternative deep learning techniques (for example prediction and times series methods) are underdeveloped. Furthermore, since most studies used supervised learning, the emerging techniques of reinforcement semi-supervised learning are lacking. Supervised learning methods are more likely to require laborious data labelling and cleaning which can affect data quality, quantity and sample size.

### Research design and potential for bias

The vast majority of included papers are retrospective cohort studies. Only 13 prospective studies were identified. There was some diversity in design with a small number of AI augmentation studies that examine the potential for AI augmentation rather than automation. The one paper that used a prospective real-world evaluation stands out for its unique method of prospective evaluation [14].

Only 5% of studies explicitly mentioned a power analysis. While it is not unusual for machine learning studies to take a “more is better” approach to sample size, this is not usually the case in the clinical radiology literature and a more robust method of sample size calculation will be needed for these methods to gain the trust necessary for clinical implementation. Hopefully, this review can serve as a reference of pilot data to estimate sample size in the future.

Ground truth is an issue that needs to be considered for all radiology research [15]. It is a challenge due to both the uncertain nature of the speciality and errors in observer performance. Only a minority of studies used pathology as the ground truth which is generally accepted as the gold standard in most clinical imaging research. In the majority of cases, the radiologic report was used as ground truth which is often a reasonable surrogate depending on the research question. A specialist report other than that of radiology and pathology was used in many cases. This has the potential to lead to errors as the inclusion of radiologists increases the quality of image interpretation [16].

Performance comparison in the machine learning literature typically involves comparing the new model to the previous state-of-the-art [17]. In clinical radiology, the standard is comparing to radiologist performance under a given set of circumstances determined by the research design [18]. It is not surprising a mix of these two methods, comparison with both human and machine performance, as seen in this review. It is significant, however, that no performance comparison was offered in 17% of cases. This design can lead to incorrect or exaggerated claims; results of such studies should be interpreted with caution. Those that compare with non-expert performance should also be interpreted cautiously as an inflated comparison could give falsely high expectations.

There is also the risk of publication bias. This is seen in the average performance metrics which are at roughly 90% of maximum performance or higher. Indeed 78% of studies that were externally validated have a performance drop of 10% or less. This high rate of high-performing studies both at internal and external validation raises the possibility that a publication bias exists [19]. Prospective registration of trials and published protocols are potential avenues for addressing this in the future.

### Data quantity and quality

There was a wide range in the quantity of data observed with papers including a range of patients in the single digits to hundreds of thousands. This serves as proof of the wide heterogeneity in the field and how each study should be interpreted on its own merits. There was a median number of 4 images per patient and of 1 case (instance in time) per patient. This is in contrast with normal clinical practice where radiologists often review upwards of a thousand images per patient across multiple points in time. Data were collected from a single existing dataset or a single hospital in the majority of cases (75%). While there was an increase in the use of multi-institutional data year-on-year, the reliance on single-centre or single repository studies limits the generalisability of reported results.

Data augmentation is commonly employed in machine learning research; however, there has been a move more recently to the use of Deep Learning to augment data in computer vision rather than simple symmetry operations [20]. While this is an interesting avenue, there have not yet been studies proving the reliability of these methods. Potential biases or errors in the data have the potential to be propagated further by these techniques. Furthermore, studies that do not use appropriate experts to label data have the potential to introduce errors [16] and reduce data quality [21].

### Explainability and Open Access

Under EU law, the GDPR gives European patients the right to explanation for all decisions made by an algorithm [22]. As such, it is a matter of concern that more than 1 in 4 studies offered no level of explanation for their outputs. We reviewed included papers for a variety of established methods of explanation [23] including examples, visualisations, natural language and counterfactuals. While cases/examples were offered as explanations in most studies, more advanced explanations such as saliency analysis and heatmaps were only seen in a minority of cases. This means that a detailed analysis of why a case was labelled as a false positive/false negative etc. would only be possible for a minority of the models provided. Modern theory points to the potential use of counterfactual examples for explanations [24] and these were not utilised by any included study.

Open data and models are one way of ensuring trust in AI models as they facilitate reproducibility [25]. It is heartening that almost half of the included studies have at least some of their data open access. The previously mentioned GDPR poses a challenge for open access data in the EU and prospective informed consent is needed to release data in many cases. The source code was only published in 14% which leaves much to be desired. While some authors may be willing to share their code on an ad hoc basis, including it with the published paper would increase accessibility and encourage more validation of results.

### From code to clinic

While it is certain that there is a delay in the implementation of AI models into clinical practise, it is also certain that radiology is leading the way. Indeed radiology accounts for 58% of medical AI devices brought to market in the USA and 53 in Europe [7]. The reasons for this include the supply demand issues outlined above. Due to the time lag between the discovery of new methods and the regulation of devices, the techniques implemented in this review may be coming to market in the near future. Indeed it is since 2015 that the rate of approval has increased, and is predicted to continue to do so [7]. There are also concerns that some devices may be used “in-house” without CE marking or FDA approval [7]. For these reasons, it is important that general radiologists are aware of the benefits and limitations of such devices as well as the scientific merit on which their claims are based. Knowledge of the literature underpinning these technical innovations is a key step in that process. Furthermore, more higher level studies with clear outcomes data are needed to show that the claims in the literature actually translate into benefits for patients. As stated in a recent review of the radiomics literature:

Carefully designed prospective, multicenter, randomized controlled trials and data sharing will be needed in the future to prove the clinical usefulness of radiomics and subsequently improved patient outcomes in a setting as close to clinical routine as possible [6]

This review has demonstrated the similar need for such higher level evidence in the in the deep learning field.

### Opportunities

The literature surrounding artificial intelligence applications radiology has exploded since 2015. However, due to a limited focus and varying quality, it is clear that opportunities remain. The focus currently is on segmentation and tumour classification. While these are important tasks that contribute significantly to radiologist workflow, interest in other areas lags. The emphasis on MRI and neuroradiology, and especially cancer imaging, means that there are opportunities in other areas. Furthermore, we have seen that “off the shelf” models, with limited or no fine-tuning, can achieve reasonable performance in these tasks in a controlled environment. As such, investigation of other tasks or implementation of these models in more general environments should be considered.

There are also opportunities for improvement in research design. Prospective registration of trials and studies that enumerate aspects of the study including sample size, ground truth, data preparation and explainability will improve the overall quality. Overall, the detail on data preparation and model optimisation was poorly reported and again the plan for these methods should be prospectively outlined (20). The quality of research design and reporting varies, and guidelines such as those issued by CONSORT [26] and the RSNA [27] should be followed to improve the quality of research overall. Furthermore, to ensure greater generalisability, the use of external validation is to be encouraged.

Within the EU, GDPR continues to pose challenges to researchers looking to maximise their quantity of data. Two clear potential avenues that could be explored further to alleviate this are the use of deep learning to create synthetic data for augmentation [28] and the process of obtaining prospective informed consent from patients for the use of their medical imaging data [29].

### Limitations

This systematic review has limitations, including publication and reporting bias. We have not included studies with unpublished data or preprint studies. We have only included papers using DL and as such papers using traditional machine learning and radiomics were excluded. Studies that do not have a clear methodology may have been misclassified. Furthermore, the heterogeneity of the included studies did not allow for meaningful meta-analysis of results. The high number of included articles only allows for a high-level overview of major themes.

## Conclusions

This review has demonstrated some of the major advances in artificial intelligence as applied to clinical radiology. It is undeniable that disruptive technologies hold promise to address the current supply/demand crisis in radiology. The consistency of performance and continued interest mean that the field continues to hold much promise. However, many published papers have varying methodological quality and a narrow focus. Furthermore, even the most promising papers often have limited potential for generalisability and clinical implementation. Many papers are at a high risk of bias, particularly due to a lack of external validation and systematic guidelines for study design are needed. A clear explanation is also lacking in the majority. Herein we have also identified many potential avenues for future research which have the potential to begin bridging the AI chasm from code to clinic.

## Supplementary Information

ESM 1 (CSV 304 kb)

## Abbreviations

AI: Artificial Intelligence
ANN: Artificial Neural Network
CONSORT: Consolidated Standards of Reporting Trials
CT: Computed tomography
CV: Computer vision
DL: Deep Learning
MRI: Magnetic resonance imaging
PRISMA: Preferred Reporting Items for Systematic Reviews and Meta-Analyses
PROSPERO: Prospective Register of Systematic Reviews
RAISE: Radiology Artificial Intelligence Systematic Evaluation of methods
RSNA: Radiological Society of North America
US: Ultrasound

## Declarations

### Guarantor

The scientific guarantor of this publication is Prof. Ronan P. Killeen.

### Statistics and biometry

No complex statistical methods were necessary for this paper.

### Methodology

• retrospective

• not applicable

• performed at one institution
