# Correction to: Radiology artificial intelligence: a systematic review and evaluation of methods (RAISE)

**DOI:** 10.1007/s00330-022-08832-1

**Published:** 2022-05-20

**Authors:** Brendan S. Kelly, Conor Judge, Stephanie M. Bollard, Simon M. Clifford, Gerard M. Healy, Awsam Aziz, Prateek Mathur, Shah Islam, Kristen W. Yeom, Aonghus Lawlor, Ronan P. Killeen

**Affiliations:** 1 St Vincent’s University Hospital, Dublin, Ireland; 2 Insight Centre for Data Analytics, UCD, Dublin, Ireland; 3 Wellcome Trust – HRB, Irish Clinical Academic Training, Dublin, Ireland; 4 School of Medicine, University College Dublin, Dublin, Ireland; 5 Lucile Packard Children’s Hospital, Stanford School of Medicine, Stanford, CA USA; 6 HRB-Clinical Research Facility, NUI Galway, Galway, Ireland; 7 Division of Brain Sciences, Imperial College London, GN1 Commonwealth Building, Hammersmith Hospital, Du Cane Road, London, W12 0HS UK

**Correction to: European Radiology**

10.1007/s00330-022-08784-6

The original version of this article, published on 14 April 2022, unfortunately contained some mistakes. The affiliations were incorrectly assigned and therefore the following corrections have been made in the original:

Brendan S. Kelly^1,2,3,4,6^

Conor Judge^3,5^

Stephanie M. Bollard^3,4^

Simon M. Clifford^1^

Gerard M. Healy^1^

Awsam Aziz^4^

Prateek Mathur^2^

Shah Islam^7^

Kristen W. Yeom^6^

Aonghus Lawlor^2^

Ronan P. Killeen^1,4^

